# Analysis of the Combined Toxic Effects of AFB1, DON, and OTA Complex Contamination in Caco-2, HK-2, SK-N-SH and HepG2 Cells

**DOI:** 10.3390/toxins18010041

**Published:** 2026-01-12

**Authors:** Hanke Zhao, Xiaohu Zhai, Weihua He, Zheng Jing, Dengyan Wang, Junhua Yang

**Affiliations:** 1 Institute for Agro-Food Standards and Testing Technology, Shanghai Academy of Agricultural Sciences, Shanghai 201403, China; zhao17337129769@163.com; 2 Institute of Pet Science and Technology, Jiangsu Agri-Animal Husbandry Vocational College, Taizhou 225300, China; zhaixiaohu@jsahvc.edu.cn (X.Z.); 2008020102@jsahvc.edu.cn (W.H.); jingzheng@jsahvc.edu.cn (Z.J.); wangdengyan@jsahvc.edu.cn (D.W.)

**Keywords:** mycotoxins, combination index, cytotoxicity, interactive effect

## Abstract

Aflatoxin B1 (AFB1), deoxynivalenol (DON), and ochratoxin A (OTA) are common mycotoxins that frequently co-occur in cereals and pose potential risks to animal and human health. This study investigated the cytotoxic effects of AFB1, DON, and OTA, individually and in binary and ternary combinations, in four human-derived cell lines representing major target organs (Caco-2, HepG2, HK-2, and SK-N-SH). Individual toxin exposure revealed cell type–dependent sensitivity, with DON generally exhibiting the strongest cytostatic effect. Combined exposure analysis showed distinct interaction patterns across cell models, including antagonistic effects of AFB1 + OTA in most cell lines, dose-dependent interactions of DON + OTA, and low-dose synergistic effects in specific combinations. Overall, the results demonstrate that mycotoxin interactions are highly dependent on dose and target cell type, and that low-dose co-contamination may enhance toxicological risks, underscoring the importance of considering combined mycotoxin exposure in health risk assessment.

## 1. Introduction

Mycotoxins are a class of low molecular mass harmful products of secondary metabolism produced by *Aspergillus*, *Penicillium* and *Fusarium*, which are the result of unregulated or technologically unsophisticated handling of grains and cereals during acquisition, storage and reprocessing [1,2,3]. In grain mycotoxins survey from 2015 to 2020 in Europe, it was observed that the most samples were simultaneously contaminated with more than one kind of mycotoxins [4]. According to the global survey of mycotoxins in pig and poultry feed, there were still considerable risks for multiple mycotoxins contamination [5], and the survey of 9000 feed samples in China from 2017 to 2021 showed that *Fusarium* toxin contamination was common, and aflatoxin and zearalenone were also presented the highly detective ratio [6]. Substantial evidence indicates that the Yangtze River Delta region in China is highly affected by AFB1, OTA, and fumonisins due to its warm and humid climate [7]. Recent studies have also reported the presence of multiple mycotoxins in children’s urine, with DON and OTA detected at the highest levels, likely reflecting high cereal consumption relative to body weight [8]. In Europe, 75–100% of agricultural products are contaminated with two or more mycotoxins, and more than 50% contain low levels of DON [9]. Consistently, our surveys detected AFB1, DON, and OTA in food and feed raw materials at 0.08–2.81, 5.00–2091.52, and 0.15–41.52 μg/kg, respectively, supporting dietary exposure to low-level mycotoxin co-contamination and its potential risk to animal and human health [10].

Accumulated data strongly suggest that these mycotoxins spread through the food chain and eventually cause health hazards to various organs of the animal or human, or even lead to death after contaminating food or feed [11]. Many recent evidences indicate that the digestion of mycotoxins can induce the cytotoxicity, carcinogenicity and other greater hazards to cells in different organs through pathways like impairing mitochondrial function, leading to oxidative stress, apoptosis, thermoapoptosis, and ferroapoptosis [2,12,13,14,15]. It has been reported that DON and AFB1 could cause the immunosuppressive effects, and increase the amount of cellular inflammatory factors [16,17]. The data in vitro showed that AFM1 or OTA exposure disrupt the intestinal microbiota balance and induce immunosuppressive effects, and DON and OTA can exert the negative effects on the kidneys and the nervous system [18,19]. Therefore, the majority previous studies have focused on the toxic effects of single mycotoxin, which indicated that AFB1 was mainly involved in the hepatotoxicity, and DON and OTA were primarily related to intestinal toxicity and nephrotoxicity, respectively [20,21,22]. However, there is fewer studies on combined toxicity of different mycotoxins, and the magnitude and type of combined toxicity of most mycotoxin combinations are less comprehensive, and more limited to binary combinations.

Recently, there has been increasing interest in combined toxic effects of different mycotoxins combination. It is indicated that the combined toxic effects such as additive, antagonistic, or synergistic can be observed when humans or animals are exposed to multiple mycotoxins after consumption or contact [2]. Some recent evidences suggested that the combined toxic effects of different toxin combinations were not uniform, and synergistic effects were observed when AFB1 and DON were simultaneously exposed to damage porcine kidney 15 cell line (PK-15) and rat hepatocytes (BRL 3A) [23,24], while antagonistic effects were occurred when combined exposure to low concentrations of AFB1 + OTA in human intestinal cells [25]. However, the majority studies have focused on the interactive effects of multiple toxins on a single kind of cell line, which was impossible to accurately reflect the toxic effects of each mycotoxin in different target organs. AFB1, OTA, and DON are the most frequently detected toxins in food safety monitoring, which are the greatest threat to agri-foods in our country [26]. Therefore, it is particularly important to comprehensively explore the combined toxicity and interactive types of these three toxins on human or animal critical organs.

In this study, AFB1, DON and OTA were used to perform separate and combined toxicity tests on human principal organ cell lines, including human hepatocellular carcinoma cells (HepG2), human renal cortex proximal tubule epithelial cells (HK-2), human colorectal adenocarcinoma cells (Caco-2) and human neuroblastoma cells (SK-N-SH). The analysis models of most common combined toxicity were as following the Bliss independence criterion and the Loewe additivity model and the median effect model proposed by Chou and Talalay [27,28]. To be more relevant to the experimental content, we chose to assay the Combination Index (CI) of the different toxin combination groups for interactive effect in each cell lines. In addition, we calculated dose reduction indices (DRI) for the evaluation of synergistic effects. By applying a unified interaction-analysis strategy across multiple cell models, this study aims to provide comparative insight into cell-type-specific interaction patterns of a common mycotoxin mixture and to contribute mechanistically grounded reference data for mixture risk assessment and alternatives to animal experimentation.

## 2. Results

### 2.1. Comparative Cytotoxic Effects of AFB1, DON and OTA on Four Types of Cell Lines

**Figure 1:**
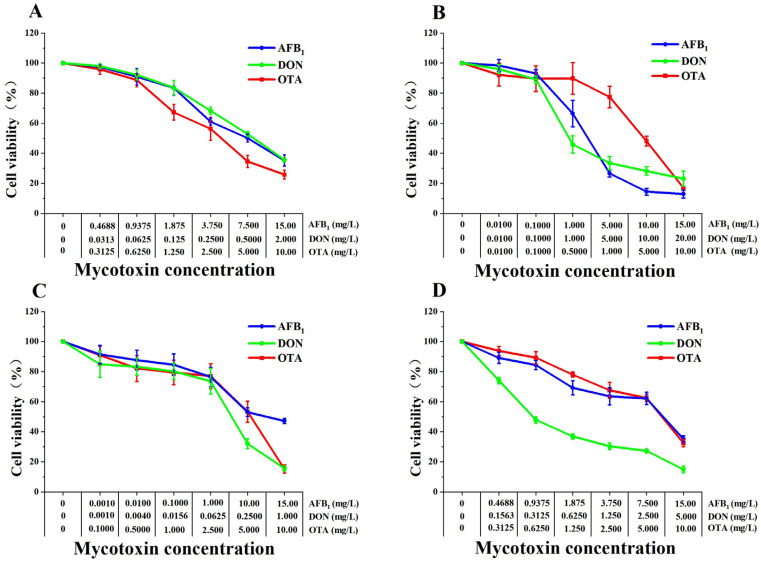
Comparative cytotoxic effects of single mycotoxins (AFB1, DON and OTA) on Caco-2, HepG2, HK-2 and SK-N-SH cells after 48 h exposure. (**A**) Caco-2 cells. (**B**) HepG2 cells. (**C**) HK-2 cells. (**D**) SK-N-SH cells. Cell viability was determined using the CCK-8 kit. Cell viability decreased with increasing AFB1, DON and OTA concentrations in a dose-dependent manner. All data are expressed as means ± SEM from six different experiments.

Cytotoxicity of AFB1, DON, OTA was assayed in Caco-2, HepG2, HK-2 and SK-N-SH cells using the CCK-8 kit and based on dehydrogenase activity in mitochondria. Following 48 h exposure to different concentrations of AFB1, DON, and OTA, the cellular viabilities were all decreased in a dose-dependent manner (Figure 1), with different half inhibitory concentration values (IC50) for each mycotoxin (Table 1). Base on the IC50 values of AFB1, the cell viability was ranking: HK-2 cells > SK-N-SH cells > Caco-2 cells > HepG2 cells; Base on the IC50 values of DON, the cell viability was ranking: HepG2 cells > Caco-2 cells > SK-N-SH cells > HK-2 cells; Base on the IC50 values of OTA, the cell viability was ranking: SK-N-SH cells > HK-2 cells > HepG2 cells > Caco-2 cells. Additionally, the cytotoxic difference of mycotoxins in the same cell line were as following: in HepG2 cells, OTA > AFB1 > DON; in Caco-2 cells, AFB1 > OTA > DON; in HK-2 cells, AFB1 > OTA > DON; in SK-N-SH cells, AFB1 > OTA > DON. As shown by the results of these four human cells, AFB1 was most sensitive to HepG2 cells, and DON and OTA were most sensitive to SK-N-SH cells and Caco-2 cells, respectively.

### 2.2. Combined Cytotoxic Effects of AFB1, DON, and OTA on Caco-2 Cells

**Figure 2:**
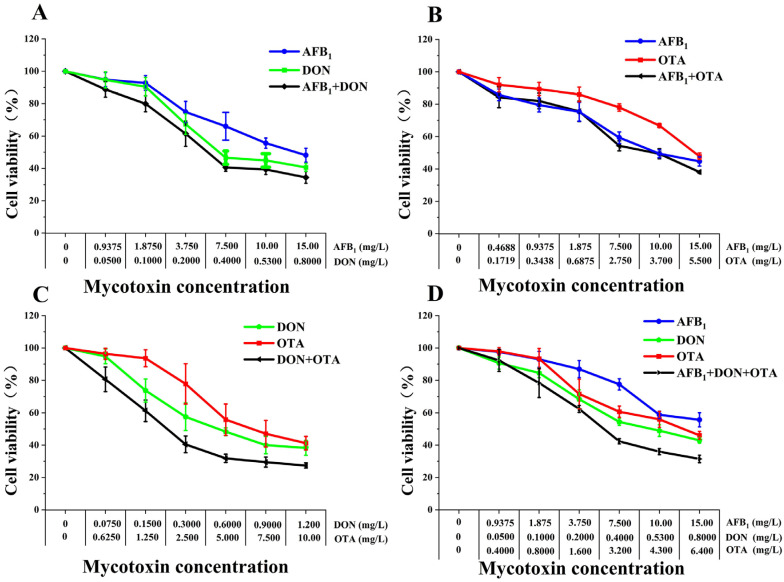
Comparative toxicities of AFB1, DON and OTA binary or ternary combinations on Caco-2 cells after 48h exposure. (**A**) AFB1 + DON. (**B**) AFB1 + OTA. (**C**) DON + OTA. (**D**) AFB1 + DON + OTA. Cell viability was determined using the CCK-8 kit. All data are expressed as means ± SEM from six different experiments. Note: The GraphPad Prism software was used to analyze the exposure of four kinds of cells to 6 concentration groups of AFB1, DON and OTA, and the IC50 value of each toxin was determined. The results are mean ± standard error. No common letter indicates differences in data (*p* < 0.05).

Following 48 h treatment to Caco-2 cells, the binary and ternary combination of AFB1, DON, and OTA on cell viability showed a dose-dependent decrease (Figure 2). At low dose levels, the cell viability in the AFB1 + OTA combined group (Figure 2B) was higher than that in a single AFB1 treatment. In contrast, the viability of cells in the AFB1 + DON (Figure 2A), DON + OTA (Figure 2C) and AFB1 + DON + OTA (Figure 2D) groups were lower than that of single mycotoxin groups

**Figure 3:**
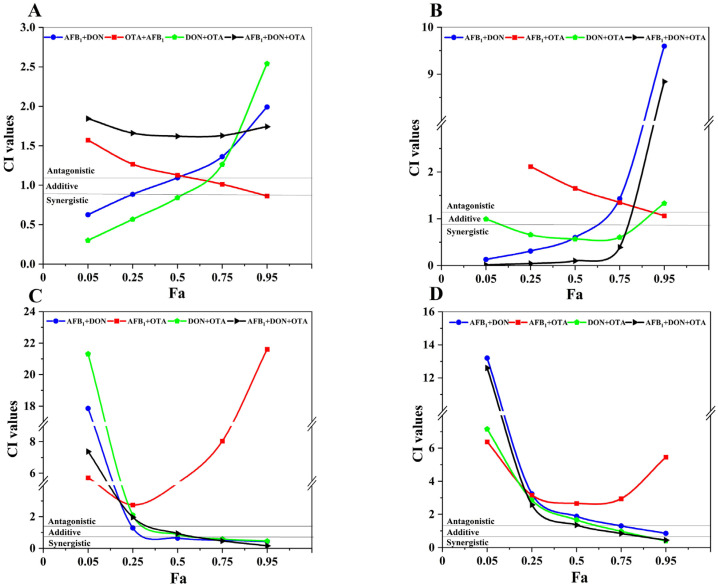
Combined index-effect curve (CI-Fa) of AFB1, DON, OTA binary and ternary combined exposure to Caco-2, HepG2, HK-2, SK-N-SH cells for 48 h. (**A**) Binary and ternary exposure of three toxins to Caco-2 cells (**B**) Binary and ternary exposure of three toxins to HepG2 cells (**C**) Binary and ternary exposure of three toxins to HK-2 cells (**D**) Binary and ternary exposure of three toxins to SK-N-SH cells. CI = 0.9–1.1, CI < 0.9, and CI > 1.1 represent additive, synergistic, and antagonistic effects, respectively. Horizontal gray solid lines correspond to lower and upper limits of the additivity zone.

In addition, the values of CIs for low (25%), medium (50%), and high (75%) cytotoxicity levels were calculated to further analysis the interactions between AFB1, DON, and OTA (95% confidence intervals) (Figure 3A and Table 2). The binary combinations of AFB1 + DON, DON + OTA showed synergistic to antagonistic effects with increasing cytotoxic effect. In contrast, the AFB1 + OTA combination showed progressively increasing additive effects (from moderate antagonism to additive). In DON + OTA combination, the synergistic effects were presented at medium and low doses, but the antagonistic effects at high doses (Table 2). To further quantify the synergistic effects of AFB1, DON, and OTA, a dose reduction index (DRI) was performed to calculate (Table 2). From the formula for calculating the DRI value, it can be seen that the greater the synergistic effect, the higher the DRI, and the higher the dose reduction multiplier (DRI multiplier) for each toxin compared to the separate group. The DRI values for the synergistic effects of AFB1 + DON and DON + OTA were decreased, and the DRI values for each toxin showed a trend from higher to lower synergistic effects, suggesting that the synergistic effects of the two combinations were higher under low cytotoxicity conditions, and that the toxicity of each toxin in combination was higher than that of each toxin separately. In addition, Caco-2 cells exposed to the ternary combination of AFB1 + DON + OTA at cytotoxicity levels > 25%, clearly identified that antagonistic effects were observed at all dose levels.

### 2.3. Combined Cytotoxic Effects of AFB1, DON, and OTA on HepG2 Cells

**Figure 4:**
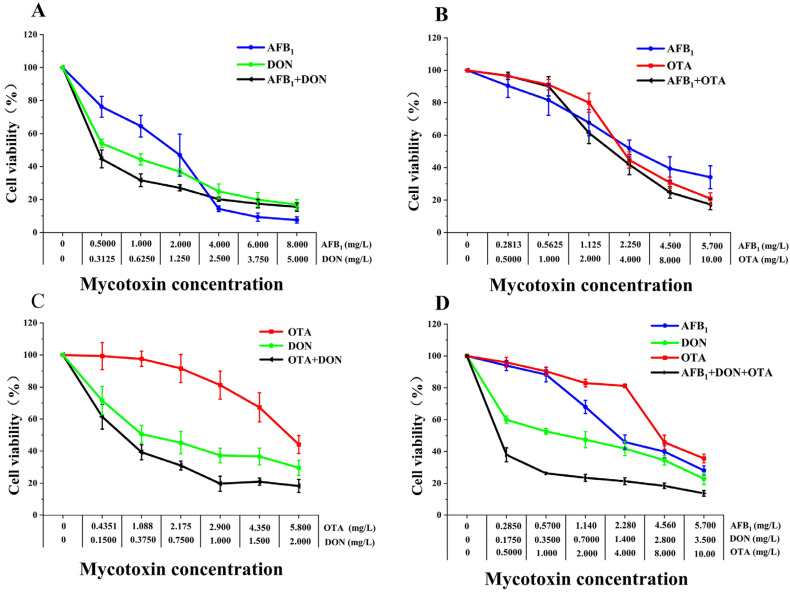
Comparative toxicities of AFB1, DON and OTA binary or ternary combinations on HepG2 cells after 48 h exposure. (**A**) AFB1 + DON. (**B**) AFB1 + OTA. (**C**) DON + OTA. (**D**) AFB1 + DON + OTA. Cell viability was determined using the CCK-8 kit. All data are expressed as means ± SEM from six different experiments.

Following 48 h treatment to HepG2 cells, the binary and ternary combination of AFB1, DON, and OTA on cell viability showed a dose-dependent decrease (Figure 4). The cell viability in the DON + OTA (Figure 3C) and AFB1 + DON + OTA (Figure 4D) combination groups was lower than that in the single mycotoxin treated groups. The AFB1 + DON (Figure 4A) combination group had lower cell viability compared with single toxin at low concentrations, but the cell viability in high concentrations were intermediate between that of the toxins alone. However, the cell viability in AFB1 + OTA (Figure 4B) group was the contrary with that in AFB1 + DON (Figure 3A) combination group.

HepG2 cells exposed to the binary combination of AFB1 + OTA at a cytotoxicity level from 25~80% showed common antagonism, and then presented the trend from addition to synergistic (Figure 3B, Table 3). With increasing levels of cytotoxicity, the AFB1 + DON and AFB1 + DON + OTA combinations exhibited a shift from synergistic to antagonistic effects. DON + OTA showed synergistic effects in this cytotoxic concentration range. Following the increase of cytotoxicity level, the DRI values of AFB1 + DON and AFB1 + DON + OTA synergistic combinations decreased and had a tendency from high to low, and showed high synergistic effects and the largest DRI values under low cytotoxicity conditions, and each toxin in the combined group was more toxic than that in the individual group; the DRI values of DON + OTA synergistic combinations appeared to be in the opposite direction, showing a change from ordinary synergism to strong synergism.

### 2.4. Combined Cytotoxic Effects of AFB1, DON, and OTA on HK-2 Cells

**Figure 5:**
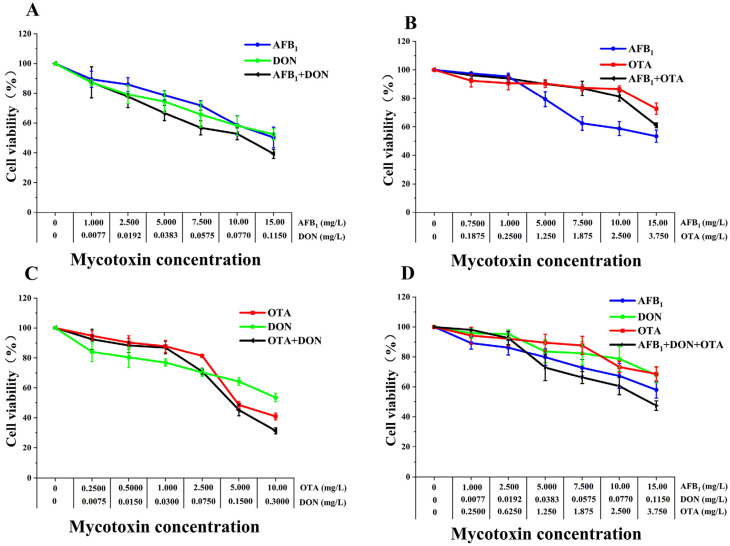
Comparative toxicities of AFB1, DON and OTA binary or ternary combinations on HK-2 cells after 48 h exposure. (**A**) AFB1 + DON. (**B**) AFB1 + OTA. (**C**) DON + OTA. (**D**) AFB1 + DON + OTA. Cell viability was determined using the CCK-8 kit. All data are expressed as means ± SEM from six different experiments.

Following 48 h treatment to HK-2 cells, the binary and ternary combination of AFB1, DON, and OTA on cell viability showed a dose-dependent decrease (Figure 5). The cell viability in the DON + AFB1 (Figure 5A) at all combination groups were lower than that in the single toxin group at different concentration ranges. At high concentrations, the cell viability of DON + OTA group (Figure 5C) was lower than that of the single toxin group. In all the concentration AFB1 + OTA combination groups (Figure 5B), the cell viability was between that of the individual toxins. In contrast, the cell viability in the high concentration AFB1 + DON + OTA (Figure 5D) group was lower than that in the individual toxin treatment, and that in the low concentration AFB1 + DON + OTA groups were between the individual toxins.

At 95% confidence intervals, the AFB1 + OTA combination showed antagonistic effect at all dose levels, whereas the AFB1 + DON +OTA combination showed synergistic effects at low concentrations, and the AFB1 + DON combination also showed the antagonistic effect at both low and medium doses. In addition, the binary combination of DON + OTA showed a synergistic to antagonistic effect at increased cellular inhibition rates observed (Figure 3C, Table 4). The DRI values associated with AFB1 + DON, DON + OTA and AFB1 + DON + OTA decreased as the cytotoxicity levels increased, showing a significant change from high to low, and the synergistic effect decreased in all conditions. From the DRI values, the dose reduction multiplicity was largest and the synergistic effect was highest at low cytotoxic dose levels for each toxin, and the toxicity of each toxin was higher in the combination group than when it was used separately.

### 2.5. Combined Cytotoxic Effects of AFB1, DON, and OTA on SK-N-SH Cells

**Figure 6:**
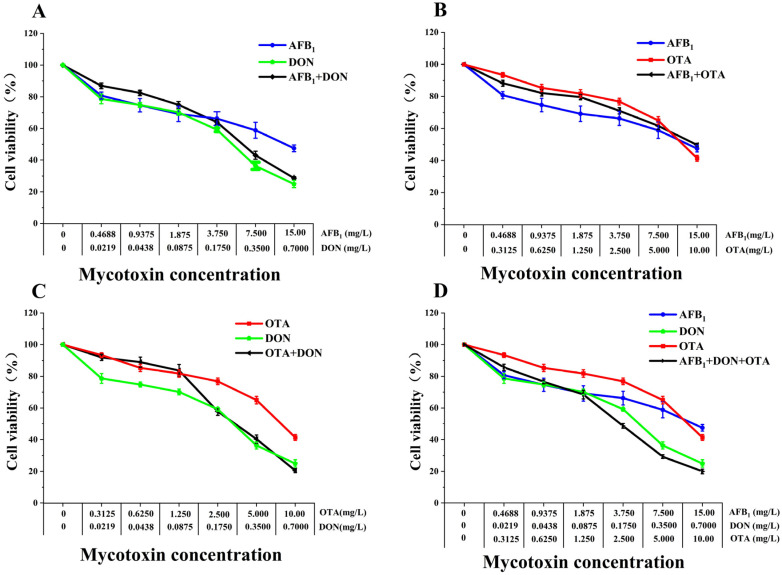
Comparative toxicities of AFB1, DON and OTA binary or ternary combinations on SK-N-SH cells after 48 h exposure. (**A**) AFB1 + DON. (**B**) AFB1 + OTA. (**C**) DON + OTA. (**D**) AFB1 + DON + OTA. Cell viability was determined using the CCK-8 kit. All data are expressed as means ± SEM from six different experiments.

Following 48 h treatment to SK-N-SH cells, the binary and ternary combination of AFB1, DON, and OTA on cell viability showed a dose-dependent decrease (Figure 6). The cell viability at the lower concentrations of AFB1 + OTA was between that of the individual toxins and the cell viability of AFB1 + DON + OTA was lower than that of the OTA group and larger than that of the AFB1 and DON groups. But at high concentrations of DON + OTA and AFB1 + DON + OTA were lower than that in the toxin alone group (Figure 6).

The interaction at high cytotoxicity levels of DON + OTA and AFB1 + DON + OTA showed proximate additive and moderated synergism, respectively. However, the interaction in AFB1 + DON, AFB1 + OTA, DON + OTA and AFB1 + DON + OTA combinations with other concentrations all showed antagonistic effects (Figure 3D, Table 5). According to the data for the three previous cells, the higher the synergistic effect, the higher the DRI, and this pattern was also consistent in SK-N-SH. In DON + OTA and AFB1 + DON + OTA, an increase in DRI, a multiplicative increase in the dose of each toxin, a higher toxicity of each toxin in the combined group than in the individual group and a change from an antagonistic to a synergistic effect were observed in both groups at high cytotoxic doses compared to low cytotoxic dose conditions.

## 3. Discussion

Based on existing studies and available data, AFB1, OTA, and DON are highly prone to co-contamination in cereals, feed, and their processed products under warm and humid environmental conditions as well as inappropriate preservation, harvesting, and storage practices. Moreover, our preliminary data indicate that these three mycotoxins frequently co-occur at low contamination levels in food and feed raw materials. Therefore, the concentration ranges applied in this study were designed to span from low-effect levels to moderate cytotoxic doses, ensuring both biological relevance and reliable dose–response analysis [10]. By exposing HepG2, Caco-2, SK-N-SH, and HK-2 to AFB1, DON, and OTA individually or in combination, we systematically evaluated the growth-inhibitory effects of these mycotoxins and their interaction patterns. The toxic effects of AFB1, DON, and OTA alone were comparatively analyzed across different cell lines, and the cytostatic effects and interaction characteristics under binary and ternary combined exposures were further determined. These results contribute to elucidating differences in target organ cell sensitivity to the three mycotoxins among the four cell models and provide a scientific basis for assessing the potential risks associated with their co-contamination in biological systems.

Cell viability was significantly reduced in all four cell types after exposure to the three mycotoxins individually or in their binary and ternary combinations. Based on the half inhibitory concentration values of each mycotoxin on different cell lines, it was determined that AFB1 toxin had the strongest inhibitory effect on HepG2 cells, and the overall inhibitory effects were HepG2 > Caco-2 > SK-N-SH > HK-2 in descending order. The inhibitory concentration of 50% of the cells exposed to HepG2 by AFB1 toxin in the present study was 1.949 mg/L, which was comparable to the inhibitory concentration of AFB1 toxin on HepG2 and HepaRG cells with IC50 of 5.995 μmol/L and 5.266 μmol/L, respectively [29]. In addition, the overall inhibitory effects of DON and OTA on the four cell types were, from highest to lowest, HK-2 > SK-N-SH > Caco-2 > HepG2 and Caco-2 > HepG2 > HK-2 > SK-N-SH, respectively. It was found that the IC50 values of 3-ADON and 15-ADON on HepG2 cells ranged from 3.6 to 6.2 μmol/L and 5.2 to 8.1 μmol/L and the lowest levels of cell survival were 44% and 40% after exposure to 10 μmol/L OTA for 48 h and 72 h, respectively, which is consistent with the half inhibition results of DON and OTA on HepG2 cells with IC50 values of 1.200 and 3.434 mg/L [30,31]. In this study, DON had the highest inhibitory effect on Caco-2 cell proliferation compared to AFB1 and OTA. The results of the study were consistent with the IC50 values in Caco-2 cells of 3.86 μmol/L, 10.8 μmol/L, and 2.33 μmol/L for 48 h of exposure to DON, 3-ADON, and 15-ADON, respectively [32]. In addition, DON showed similar low dose exposure high cytostatic effect on HK-2 and SK-N-SH. The low dose exposure high cytostatic effect produced by DON on the four cells might be a result of the multiple inhibitory effects of DON on a number of different targets in eukaryotic cells such as protein inhibition, DNA and RNA synthesis as well as mitochondrial inhibitory functions [33]. In this study, the IC50 value of OTA for Caco-2 was similar to that of 21.25 μmol/L for OTA-exposed Caco-2 cells for 24 h [34]. However, the difference in IC50 values of OTA among the four cells was not significant, which might be related to the different cell culture methods, reagents and cell line types.

And the Chou-Talalay method to study the binary and ternary interactions of AFB1, DON and OTA toxins has been widely used to determine whether the cytotoxicity induced by a combination of mycotoxins was greater than, equal to, or less than the expected. Determination of the interaction of AFB1, DON and OTA multiple exposures on HepG2, Caco-2, SK-N-SH and HK-2 cells by analysing CIs and DRIs at different cytotoxicity levels [28]. Dose changes in the combination of AFB1, DON and OTA were correlated with the type of interaction observed. However, the interaction analysis in this study is based solely on cell viability, primarily reflecting overall interaction trends rather than definitive biological mechanisms.

Exposure to the AFB1 + DON combination resulted in broadly comparable interaction trends in HepG2 and Caco-2 cells, characterized by a tendency toward synergistic effects at lower cytostaticity levels and antagonistic effects at higher cytostaticity levels. In HK-2 cells, synergistic interactions were observed at 25% and 50% cytostaticity, whereas antagonistic effects predominated at 75% cytostaticity. In contrast, SK-N-SH cells showed predominantly antagonistic interactions across most cytostaticity levels. Although no mechanistic endpoints were assessed in the present study, these cell-type–dependent interaction patterns may be interpreted in light of previously reported modes of action. AFB1 is known to induce DNA damage through the formation of DNA adducts, whereas DON primarily interferes with ribosomal function, leading to inhibition of protein synthesis and reduced cell proliferation. The concurrent disruption of DNA integrity and protein synthesis has been suggested to enhance cytotoxic outcomes in metabolically active cells such as hepatocytes and intestinal epithelial cells, which may partly account for the low-dose synergistic trends observed in HepG2 and Caco-2 cells. Consistent with this notion, chronic exposure studies in piglets fed DON-contaminated diets (0.9–4 mg/kg) have reported oxidative stress, increased intestinal permeability, and inhibition of protein synthesis and cell proliferation, effects that may contribute to dose-dependent modulation of combined toxicity [35]. In addition, exposure to AFB1 and DON adducts exerted a significant inhibitory effect on the proliferation of glial cells, and as the brain was an important target organ for associating neurotoxicity and neurodegenerative diseases [36], the mycotoxin contamination might be a key factor contributing to the neurotoxicity and dysfunction of glial cells. The AFB1 + OTA combination exhibited a dose dependent interactive trend in Caco-2 cells, primarily showing antagonistic effects at low to moderate levels of cellular inhibition, while synergistic effects emerged at higher inhibition levels, and antagonistic effects at all dose groups when exposed to HepG2, HK-2 and SK-N-SH cells. The interaction effects of the DON + OTA combination exhibited marked cell type and dose dependence. At low and moderate inhibition levels in HK-2 and Caco-2 cells, a synergistic effect was observed, whereas at high inhibition levels in Caco-2 cells and in SK-N-SH cells, the combination primarily produced antagonistic or additive effects. Regarding HepG2, in contrast, AFB1 + OTA had a slight synergistic/proximate additive effect; furthermore, OTA + DON had a moderate antagonistic/proximate cumulative effect [25]. The results of the present study were in line with pervious study, suggesting that AFB1, DON and OTA might induce the underlying mechanisms, such as inducing apoptosis to inhibit cell proliferation or promoting cell death through oxidative stress, affecting cell proliferation or promoting cell death in cells through certain common mechanisms. The results of this study could be explored more deeply and be important in elucidating how AFB1, DON and OTA collectively damage human health by affecting cells of vital organs, in order to reduce the risk of mycotoxins to human health and to ensure the safety of food of animal origin.

## 4. Conclusions

In the present study, we used multiple human organ cells as a model to determine the variable effects of mycotoxins individually and in combination. The inhibitory ranking of the three toxins exposed to various cells according to the half inhibitory concentration value (IC50). DON showed the highest cytostatic activity in lower doses against HepG2, Caco-2, SK-N-SH and HK-2 cells, and AFB1 showed targeted toxicity against HepG2. However, the effects of mycotoxin combinations cannot be predicted solely on the basis of individual components. The combined effects of binary and ternary combination of AFB1, DON and OTA on HepG2, Caco-2, SK-N-SH and HK-2 were different due to the change of toxin dosage and different cell types. The observed synergistic effects of DON + OTA at low doses in HepG2, Caco-2 and HK-2 cells, especially at low doses in food and animal feed, would pose a significant threat to intestinal and immune health. It was also observed that different combinations of concentrations of AFB1 + DON showed low-dose synergism and high-dose antagonism on HepG2 cells, which mean that their co-contamination was not only hepatotoxic, but also appeared to exacerbate the toxic effect. The mechanism of toxicological interactions between AFB1 and DON was not explored in this study and needs to be further investigated, which might provide new insights to reduce the hazard of combined AFB1 and DON pollution.

## 5. Materials and Methods

### 5.1. Chemicals

Certified standards of AFB1, DON and OTA with purity > 99% were purchased from Pribolab Biological Technical Company (Pribolab, Singapore). Phosphate buffer saline (PBS) solution (pH = 7.0) and penicillin-streptomycin (10,000 Units/mL-10,000 μg/mL) were purchased from Beijing Solarbio Science and Technology Co., Ltd. (Beijing, China). 0.25% Trypsin-EDTA solution was purchased from GIBCO and stored at −20 °C (Shanghai, China). Minimum Essential Medium (MEM) and fetal bovine serum (FBS) were kindly provided by Procell Life Science and Technology Co., Ltd. (Shanghai, China). Dimethyl sulfoxide (DMSO) was purchased from Shanghai Enzyme-linked Biotechnology Co., Ltd. (Shanghai, China), and Cell Counting Kit-8 (CCK-8) was supplied by Shanghai BioScience Co., Ltd. (Shanghai, China). All other chemicals, unless specified, were purchased from Sigma-Aldrich Pte. Ltd. (Saint. Louis, MO, USA).

### 5.2. Cell Culture

Human cell lines HepG2 (CL-0103), HK-2 (CL-0109), and SK-N-SH (CL-0214) were purchased from Procell Life Science and Technology Co., Ltd. (Shanghai, China). Caco-2 (JN2042S) was purchased from AnWei-sci. (Shanghai, China). These cell lines were selected as well-established in vitro models representing major target organs of mycotoxin exposure, including the liver (HepG2), intestine (Caco-2), kidney (HK-2), and nervous system (SK-N-SH), and are widely used in mycotoxin toxicology studies. All cell lines were cultured in 75 cm^2^ flasks containing Minimum Essential Medium (MEM) supplemented with 10% fetal bovine serum (FBS) and 1% penicillin–streptomycin, and maintained at 37 °C in a humidified incubator with 5% CO_2_. For passaging, cells were washed with PBS, detached using 0.25% trypsin–EDTA for 3 min, neutralized with complete medium, collected by centrifugation at 960× *g* for 5 min, and resuspended in fresh culture medium for subsequent experiments.

### 5.3. Mycotoxin Exposure Design for Individual and Combined Treatments

At the beginning of the experiment, AFB1, DON, and OTA were dissolved in methanol, purified water, and dimethyl sulfoxide (DMSO) to prepare stock solutions at concentrations of 100 g/L, 2 g/L, and 100 g/L, respectively. These stock solutions were further diluted with culture medium to obtain working solutions, with final solvent concentrations maintained below 1.5% for methanol and 0.1% for DMSO, levels at which no effects on cell viability were observed. Cells were seeded into 96-well plates at a density of 8 × 10^3^ cells per well and allowed to attach for 24 h at 37 °C. Subsequently, cells were exposed to individual mycotoxins as well as binary and ternary combinations at various concentrations. The final concentration ranges of AFB1, DON, and OTA spanned from 0.001 to 20 mg/L, and the specific concentration gradients and combination ratios—designed based on the IC_50_ values of each mycotoxin—are summarized in Table 6. Control groups were incubated with culture medium only, while blank wells contained culture medium without cells. All treatments were performed with six replicates.

### 5.4. Cell Viability Assay

Cells were diluted to a density of 8 × 10^3^ cells/well and seeded in 96-well culture plates. After 24 h incubation, all cells were exposed to various concentrations of individual or combined contaminants and allowed to grow in an incubator for 48 h, respectively. The cytotoxicity of the two different cell lines was determined using a CCK-8 kit according to the manufacturer’s instructions. After treatment, 10 μL CCK-8 solution was added to each well (10% culture volume) and incubated for 2 h at 37 °C. Absorbance values (A) were measured at 450 nm using a microplate reader (Multiskan Sky, Thermo, Singapore). All treatments were performed in six replicates, and cell activity was calculated as follows:

$$Cell\;Viability\;(\%)=\frac{A\left(experiment\;\right)-A(blank)}{A\left(control\right)-A(blank)}\times \;100$$

### 5.5. Combined Index Analysis of Mycotoxin Mixtures

The dose-effects and the CI for multiple exposures of AFB1, DON and OTA in different cells were calculated according to the mass action law of the median effect equation [37]:

$$\frac{f_{a}}{f_{u}}={\left(\frac{D}{Dm}\right)}^{m}$$

*D* is the mycotoxin concentration, *f_a_* is the cell proliferation inhibition rate, and *f_u_* is the unaffected fraction (*f_u_* = 1 − *f_a_*). *D_m_* is the median effect dose for the 50% inhibition of cell proliferation (m is the coefficient indicating the shape of the dose-effect relationship (*m* = 1, *m* > 1, and *m* < 1 each indicate hyperbolic, sigmoidal, and flat sigmoidal dose-effect curves). The method employs the *D_m_* parameter to measure the cytotoxicity of individual mycotoxins.

When the composition of toxins extends to binary or multivariate, the interactions of multiple mycotoxins were assayed by means of CI approach based on the median effect principle (MEP), as the CI values calculated according to a generic formula:

$${n_{\left(CI\right)}}_{x}=\sum_{j=1}^{n}\frac{{\left(D\right)}_{j}}{{\left(D_{x}\right)}_{j}}$$

*n* (*CI*) *x* refers to *n* kind of toxin combined cell inhibition rate for *x*% of composite index, (*D*)_*j*_ is refers to the joint action of *n* kind of toxin to total dose of *x*% cell inhibition rate, and (*D*_*x*_)_*j*_ refers to the combination of each toxin effect itself when dose of *x*% cell inhibition rate. Overall, the types of joint effects represented by CI = 0.9–1.1, CI < 0.9 and CI > 1.1 were additive, synergistic and antagonistic, respectively. The specific classification criteria and corresponding symbols were shown in Table 7.

Dose reduction indices (DRI), which were correction factors for quantifying synergistic interactions based on the occurrence of synergistic effects in binary or ternary mixtures, were also calculated by CompuSyn software. The DRI indicated how many times the dose of each toxin in the combination decreased when the combined toxins reached *x*% cell inhibition compared to the dose of each toxin alone that reached *x*% cell inhibition. The *DRI* for each toxin in the combination was calculated using the following formula:

$$n_{{\left(CI\right)}_{x}}=\sum_{j=1}^{n}\frac{{\left(D\right)}_{j}}{{\left(D_{m}\right)}_{j}}=\sum_{j=1}^{n}\frac{1}{{\left(DRI\right)}_{j}}$$

### 5.6. Statistical Analysis

GraphPad Prism 9.4.0 software was used to calculate the IC50 values of the three different mycotoxins exposed to the four cell lines. All data were assayed by ANOVA using IBM SPSS Statistics 20 software, LSD significance test, *p* < 0.05 for significant difference, *p* < 0.01 for highly significant difference, and all the data of the results were expressed as mean ± SEM. Origin 2021 was used for performing the line plotting. ComboSyn 3.0.1 software (Inc., Paramus, NJ, USA) was used for calculating the indexes of the CI and DRI about three toxins.

## Figures and Tables

**Table 1:** IC50 values of four human-derived organoids exposed to AFB1, DON and OTA for 48 h (in 95% confidence interval).

Mycotoxins	Caco-2 Cells		HepG2 Cells		Hk-2 Cells		SK-N-SH Cells	
	IC50	Ratio vs. AFB1	IC50	Ratio vs. AFB1	IC50	Ratio vs. AFB1	IC_50_	Ratio vs. AFB_1_
AFB_1_ (mg/L)	8.33 ± 0.49 ^a^	1	1.95 ± 0.63 ^b^	1	17.65 ± 4.68 ^a^	1	8.82 ± 1.84 ^a^	1
DON (mg/L)	0.65 ± 0.06 ^c^	0.08	1.20 ± 0.20 ^b^	0.62	0.14 ± 0.05 ^c^	0.01	0.39 ± 0.04 ^c^	0.05
OTA (mg/L)	3.07 ± 0.79 ^b^	0.37	3.43 ± 1.28 ^b^	1.76	4.49 ± 1.27 ^b^	0.25	5.94 ± 0.69 ^a^	0.67

Data are presented as the means ± SEM, *n* = 6 (six experiments). ^a^, ^b^ and ^c^: in each column, values without a common letter differ significantly (*p* < 0.05).

**Table 2:** CI, DRI values and types of interaction after binary and ternary combined exposed to AFB1, DON, OTA on Caco-2 (in 95% confidence interval).

Cell Types	Toxin Combinations	Combination Ratio	25% Cytotoxicity			50% Cytotoxicity			75% Cytotoxicity		
			CI Values	DRI		CI Values	DRI		CI Values	DRI	
Caco-2	AFB1 + DON	1:0.08	0.88	2.82	++	1.09	2.50	±	1.36	\	--
				1.89			1.44			\	
	AFB1 + OTA	1:0.37	1.27	\	--	1.11	\	-	1.01	1.45	±
				\			\			2.89	
	DON + OTA	1:8.33	0.57	2.78	+++	0.84	2.03	++	1.26	\	--
				4.78			2.85			\	
	AFB1 + DON + OTA	1:0.05:0.42	1.66	\	---	1.62	\	---	1.63	\	---
				\			\			\	

**Table 3:** CI, DRI values and types of interaction after binary and ternary combined exposed to AFB1, DON, OTA on HepG2 (in 95% confidence interval).

Cell Types	Toxin Combinations	Combination Ratio	25% Cytotoxicity			50% Cytotoxicity			75% Cytotoxicity		
			CI Values	DRI		CI Values	DRI		CI Values	DRI	
HepG2	AFB1 + DON	1:0.007	0.31	19.32	+++	0.60	4.85	+++	1.43	\	--
				3.87			2.53			\	
	AFB1 + OTA	1:0.25	2.11	\	---	1.6481	\	----	1.34	\	----
				\			\			\	
	DON + OTA	1:33.33	0.66	2.88	+++	0.5680	2.17	+++	0.60	1.6366	+++
				3.89			9.30			22.252	
	AFB1 + DON + OTA	1:0.007:0.41	0.04	384.02	+++	0.10	47.92	+++	0.39	5.9808	+++
				27.04			14.59			7.8770	
				640.39			82.35			10.591	

**Table 4:** CI, DRI values and types of interaction after binary and ternary combined exposed to AFB1, DON, OTA on HK-2 (in 95% confidence interval).

Cell Types	Toxin Combinations	Combination Ratio	25% Cytotoxicity			50% Cytotoxicity			75% Cytotoxicity		
			CI Values	DRI		CI Values	DRI		CI Values	DRI	
HK-2	AFB1 + DON	1:0.63	0.51	829.91	+++	0.64	1.84	+++	1.29	\	--
				26.31			9.50			\	
	AFB1 + OTA	1:0.56	8.04	\	---	4.50	\	---	2.74	\	--
				\			\			\	
	DON + OTA	1:2.9	0.59	267.08	+++	0.88	3.29	+	2.10	\	---
				31.88			1.72			\	
	AFB1 + DON + OTA	1:0.61:1.75	0.46	26.23	+++	0.94	2.45	±	1.97	\	---
				20.69			3.04			**\**	
				2.01			2.92			**\**	

**Table 5:** CI, DRI values and types of interaction after binary and ternary combined exposed to AFB1, DON, OTA on SK-N-SH (in 95% confidence interval).

Cell Types	Toxin Combinations	Combination Ratio	25% Cytotoxicity			50% Cytotoxicity			75% Cytotoxicity		
			CI Values	DRI		CI Values	DRI		CI Values	DRI	
SK-N-SH	AFB1 + DON	1:0.05	3.24	\	---	1.88	\	---	1.30	\	--
				\			\			\	
	AFB1 + OTA	1:0.67	3.16	\	---	2.66	\	---	2.95	\	---
				\			\			\	
	DON + OTA	1:14.28	2.87	\	---	1.67	\	---	0.97	1.39	±
				\			\			3.92	
	AFB1 + DON + OTA	1:0.05:0.67	2.58	\	---	1.36	\	--	0.84	13.39	++
				\			\			1.76	
				\			\			4.95	

**Table 6:** Concentration ranges of AFB1, DON, and OTA used for cell viability assays.

Cell Line	Toxin	Concentrations (mg/L)
HK-2	AFB1	0.001, 0.01, 0.1, 1, 10, 15
	DON	0.001, 0.004, 0.0156, 0.0625, 0.25
	OTA	0.1, 0.5, 1, 2.5, 5, 10
Caco-2	AFB1	0.3125, 0.625, 1.25, 2.5, 5, 15
	DON	0.0313, 0.0625, 0.125, 0.25, 0.5, 2
	OTA	0.3125, 0.625, 1.25, 2.5, 5, 10
HepG-2	AFB1	0.001, 0.1, 0.5, 1, 5, 10, 15
	DON	0.01, 1, 5, 10, 20
	OTA	0.01, 0.1, 0.5, 1, 5, 10
SK-N-SH	AFB1	0.4688, 0.9375, 1.875, 3.75, 7.5, 15
	DON	0.1563, 0.3125, 0.625, 1.25, 2.5, 5
	OTA	0.3125, 0.625, 1.25, 2.5, 5, 10

**Table 7:** Descriptions and symbols of the degrees of combined toxicity grading.

Combination Index Value	Description	Graded Symbols
<0.10	Very strong synergism	+ + + + +
0.10~0.30	Strong synergism	+ + + +
0.30~0.70	Common synergism	+ + +
0.70~0.85	Moderate synergism	+ +
0.85~0.90	Slight synergism	+
0.90~1.10	Nearly additive	±
1.10~1.20	Slight antagonism	−
1.20~1.45	Moderate antagonism	− −
1.45~3.30	Common antagonism	− − −
3.30~10.0	Strong antagonism	− − − −
>10.0	Very strong antagonism	− − − − −

## Data Availability

The original contributions presented in this study are included in the article. Further inquiries can be directed to the corresponding author.
